# Influence of Salicylic Acid on the Excretion of Uric Acid

**Published:** 1889-01

**Authors:** 


### INFLUENCE OF SALICYLIC ACID ON THE EXCRETION OF URIC ACID.

A year and a half ago Mr. A. Haig read a paper before the Royal Medical and Chirurgical Society of London in which he drew attention to the relation of a certain form of headache to the excretion of uric acid, showing that the headache in question coincided with a plus excretion of uric acid, and was possibly due to an excess of this acid in the blood. In a later paper, in the *Journal of Physiology*, Mr. Haig showed that alkalies, as soda and potash, always increase the excretion of uric acid, within certain limits, while acids such as muriatic, citric, and acetic, always diminish the excretion of uric acid. The contrary is true of salicylic acid, however; and it is now shown that this drug is of very great value in rheumatism and gout.

The action of salicylic acid is to dissociate the excretion of uric acid from its general relations to acidity, so that an increase of acidity, while there is plenty of salicylate in the blood, is accompanied by no diminution of uric acid excretion. It is known that acids play an important role in dyspepsia, gout and rheumatism; and if the salicylates will prevent these acids causing retention of uric acid, we can easily explain a large part of their action in these affections. So high an authority as Sir Alfred Garrod makes the distinct statement that "when a few glasses of wine, ale, or porter quickly and invariably produce in an individual an inflammatory affection of a joint, such inflammation is of a truly gouty character"; and there is little or no doubt that this gouty inflammation is due to hypersecretion of uric acid.

From Mr. Haig's experiments salicylate of soda does not appear to have any definite effect on the acidity of the urine, but it is seen that the uric acid secretion is not influenced by the acidity in the usual way. It is shown, in a paper read by Mr. Haig before the Royal Medical and Chirurgical Society a year ago, and published in the *Medico-Chirurgical Transactions*, Vol. 53, that under salicylate, uric acid excretion is not kept down by rise in acidity, but rather the reverse; and that a large excess of uric acid excretion, when it occurs under salicylate, is not accompanied by headache. To these facts must be added several others: Brunton has shown that salicylate of soda is a valuable remedy in headache; we know also that some form of salicylate is a relief to the pains of acute rheumatism, and prevents or relieves the pains and discomforts of some phases of gout. We know again that it is the headache of persons with a gouty tendency that is most frequently and easily relieved by salicylate, and it is reasonable to infer that the headache relieved by salicylate is an expression of so-called "quiet gout." Thus it appears that these headaches are due to hypersecretion or excretion of uric acid.

If we accept the statement of Latham that salicylic acid combines with glycocine and passes out in the urine as salicyluric acid, and if we suppose that salicylic acid has the power of splitting up uric acid and combining with its glycocine so as to form salicyluric acid (as benzoic acid does to form hippuric acid), we are on the road to an explanation of the reversal by salicylate of the usual action of acidity on the excretion of uric acid. What we know of the chemistry of salicyluric acid leads us to suppose that it is more soluble than uric acid, both in the tissue fluids and in slightly acid solutions; peculiarities that are sufficient to explain the altered relation of uric acid excretion to acidity under salicylates. Both hippuric and salicyluric acid, there is reason to believe, are far more soluble in water than is uric acid, and also more soluble in water containing hydrochloric acid. Now, if when we administer salicylate the uric acid in the blood and tissue fluids is converted into salicyluric acid, we have to deal with a substance far more soluble in those fluids than is uric acid, and also more soluble in slightly acid fluids; and if change of solubility be the true explanation of the action of acids and alkalies on uric acid excretion, the results obtained with the salicylates—that a rise in acidity no longer entails a fall in uric acid excretion—are easy of explanation.

Mr. Haig believes that, as in the case of alkalies, salicylic acid acts on the uric acid supposed to be stored in the liver and spleen, and, its solubility being increased, it rushes at once into the blood and out of the system through the kidneys. This rush into the blood under the action of the salicylates is not accompanied by headache, thus differing from the rush under the alkalies; so that there are reasons for believing that the chemical combination in the blood of uric acid and the salicylates is different from that formed between uric acid and alkalies.

In a paper read before the same society last April, Mr. Haig contrasts the above-mentioned action of the salicylates with that of several drugs that cause retention of uric acid. It is shown that lead, taken as the acetate, causes clearly and distinctly retention of uric acid. Iron, also, causes retention of uric acid, though not so quickly as the lead salt. Sir Alfred Garrod says that urates of lead and iron are quite insoluble, which fact may explain their action in causing retention. Lithium and the lithia salts have enjoyed a high reputation for several years in gouty and rheumatic affections—in the uric acid diathesis, though they have, unexpectedly and in an unwelcome manner, shown themselves completely inert in many cases. Its urate is extremely soluble, but from Mr. Haig's experiments there appears to be no doubt that it causes retention of uric acid. Mr. Ure pointed out in 1860 that lithia was no use as a solvent of uric acid when taken by the mouth. Carbonate of lithia had been recommended in the treatment of vesical calculus; but Mr. Ure pointed out that it would undergo decomposition and precipitation when coming in contact with ammonia-phosphate of soda, and thereby be rendered inert. Given internally it forms a nearly insoluble triple phosphate with phosphates of acids, or with the triple phosphates of ammonia and soda—salts that are generally present in animal fluids. Lithia taken internally, therefore, never reaches the uric acid to form a soluble urate of lithia, but, on the other hand, by forming an insoluble compound with phosphate of soda, it practically removes from the blood one of the well-known solvents of uric acid. If it be desired to give lithia for any special reason, one-drachm doses of sodium phosphate should be given three times a day, in order to supply the place of the sodium phosphate removed by combination with the lithia, thus preventing the lithia causing any retention of uric acid. But it is just as well not to use lithia.

The connection between lead-poisoning and epilepsy has been regarded as rather mysterious. But knowing that lead causes retention of uric acid, and that epilepsy may be caused by retention of uric acid, the apparent mystery is cleared up. Some cases of epilepsy yield uric acid reactions identical with those obtained in some cases of headache; and if some headaches are due to uric acid retention, it is likely that some epileptic attacks are also due to such retention, and can be prevented like the headaches by treatment directed to the uric acid factor. There is a distinct clinical relationship of epilepsy both to headache and gout. All epileptic fits are not due to uric acid; but it is probable that it will be shown that the cases of epilepsy that have a marked family-history of gout, rheumatism, headache, or epilepsy, will give uric acid reactions, and may be cured by treatment directed to the uric acid factor, as in the case of the corresponding headache. It is in these cases of epilepsy that lead and iron will do harm, and the salicylates may be expected to be beneficial.

As may be seen, Mr. Haig's suggestions are far from being empirical, and the field that he has opened is certainly worth the working. It is certainly a significant fact that the vegetable diet that Mr. Haig has found so useful in headaches due to uric acid has been found most beneficial in some cases of epilepsy. His suggestions are partly borne out by Radcliffe's observation, that by giving iodide of potassium and bicarbonate of potassium with bromide, he was able to give less bromide in cases of epilepsy. Radcliffe is very emphatic as to the disadvantages of associating iron with drugs given in epilepsy.

